# Reducing Barriers to Postabortion Contraception: The Role of Expanding Coverage of Postabortion Care in Dar es Salaam, Tanzania

**DOI:** 10.9745/GHSP-D-19-00146

**Published:** 2019-08-22

**Authors:** Benjamin Stephens, Isihaka Jossey Mwandalima, Amani Samma, Jean Lyatuu, Kathryn Mimno, Joseph Komwihangiro

**Affiliations:** a Pathfinder International, Watertown, MA, USA.; b Pathfinder International, Dar es Salaam, Tanzania.

## Abstract

**Background:**

In Tanzania, limited access to postabortion care (PAC) contributes to high rates of maternal mortality. To address the issue, Pathfinder International and the Tanzania Ministry of Health, Community Development, Gender, Elderly and Children (MOHCDGEC) introduced and expanded coverage of PAC in 64 public health facilities in Dar es Salaam, Tanzania.

**Methods:**

During a 30-month period, we implemented a multifaceted approach to introduce and expand PAC, including clinical training and mentorship for health care providers; service reorganization, equipment provision, and an expanded method mix offering; standardization of PAC reporting tools; and community engagement and referral. We assessed outcomes using PAC service statistics from 64 public health facilities in 4 districts of Dar es Salaam and health care provider mentorship data from 385 observed PAC visits.

**Results:**

From January 2016 to June 2018, voluntary postabortion contraceptive uptake increased steadily. A total of 6,636 PAC clients, including 2,731 young people (ages 10–24), adopted a method post-procedure. Average semesterly client volume per facility increased from 27 to 52.4 manual vacuum aspiration clients and 17.6 to 43.9 postabortion contraceptors between the first and last periods. Overall postabortion contraceptive uptake was 80.6% (6,636/8,230), with a method mix of 58.3% implant, 18.9% intrauterine device, 13.7% pills, 8.6% injectables, and 0.5% permanent methods. Adults and young people had comparable method mix. Mentored providers showed improvements in service quality indicators. During the last period, 92% counseled the client on contraception, 93% considered the client’s sexual and reproductive health intentions, 94% provided correct method information and supply, and 96% documented services on the client’s family planning card. Different provider types (mid- and senior-level) performed comparably.

**Conclusions:**

Expanding PAC coverage to primary- and secondary-level facilities led to high uptake of voluntary contraception among postabortion clients. Key interventions included PAC clinical training and mentorship; service reorganization, equipment provision, and an expanded method mix offering; use of standardized PAC registers; and community engagement for awareness building and linkage to PAC.

## Abstract (precis)

Expanding postabortion care (PAC) coverage to 64 public facilities over 30 months in Dar es Salaam, Tanzania, contributed to >6,000 women voluntarily adopting a contraceptive method, for an overall acceptance rate of about 81% and 78% adopting a long-acting method. Key interventions included clinical training and follow-up mentorship; PAC service reorganization, equipment provision, and an expanded method mix offering; standardized PAC documentation tools; and community linkages and referrals.

## BACKGROUND

Treatment of complications resulting from unsafe abortions and prevention of unintended pregnancies are essential strategies for reducing maternal mortality, which globally claims approximately 830 lives each day.^1^ Yet many countries face challenges implementing such strategies, despite national and global efforts to support them. Sub-Saharan Africa disproportionally experiences high maternal death rates resulting from unsafe or untreated abortions, which accounted for 9.6% of all maternal deaths in the region between 2003 and 2009.^2^ In Tanzania, the maternal mortality ratio is estimated to be 556 deaths per 100,000 live births,^3^ and despite concerted efforts in recent years, the country still struggles to reduce the number of deaths. While Tanzania has improved child survival rates due to sustained investments over time and a focus on high-impact practices at lower levels of the health system, maternal health has had more variable commitments, which have occurred at higher levels of the health system and been implemented at a smaller scale.^4^ Abortion is restricted in Tanzania and unsafe abortion plays a substantial role in Tanzania’s poor maternal health performance, with an annual incidence of induced abortions estimated at 405,000.^5^ The problems are compounded by limited access to contraceptive services, which leads to approximately 1 million unintended pregnancies annually^6^: the national modern contraceptive prevalence rate is only 27%, while 22% of the women who wish to delay or limit childbearing are not using modern contraception.^3^

High-quality postabortion care (PAC) can greatly help reduce the morbidity and mortality associated with unsafe or incomplete abortions, and it includes both curative and preventive carecomponents.^7^ Experts have identified 5 essential, interrelated components of PAC: treatment of complications, counseling, contraceptive services, other sexual and reproductive health (SRH) services such as sexually transmitted infection (STI) testing and treatment, and community partnerships and service linkages.^8^ Research has demonstrated the importance of information provision and linkages to ancillary services—including comprehensive counseling and contraception—as part of the PAC service package, and it has shown them to be recognized indicators of quality care.^9^^,^^10^ An expanded method mix is another indicator of quality care. A recent multicountry analysis identified the provision of at least 1 modern, short-acting contraceptive method as a signal function of a health system’s capacity to provide basic PAC and provision of at least 1 short-acting and 1 long-acting reversible contraceptive (LARC) method or permanent method as signal functions for comprehensive PAC capacity.^11^

Decades of program implementation have generated evidence and contributed to globally recognized best practices for postabortion contraception. Contraceptive counseling and full method availability at the same time and location where women receive treatment for abortion complications is a proven high-impact practice because it makes contraception available immediately after the procedure.^12^ Removal of cost barriers by offering free contraceptive methods and services, including LARCs, further contributes to contraceptive uptake.^13^ Practitioners have made other essential recommendations for increasing uptake of postabortion contraception among vulnerable groups such as youth and adolescents, including the removal of facility policies and practices that require women to be married or have parental or spousal consent for contraception and the presence of skilled providers trained in nonjudgmental and respectful youth-friendly service delivery.^14^

A correlation exists between improving the quality of postabortion care services and increasing the uptake of postabortion contraception. Experience in Togo shows that even within a relatively short period, contraceptive counseling and method uptake among postabortion clients increased substantially when PAC service quality improved. Mugore and colleagues claimed that^15^:

high-quality PAC services avert repeat unplanned pregnancies and the cycle of repeat abortions; they do this by providing counseling and a broad range of contraceptive services at the time and location of emergency treatment of abortion complications, and before the patient is discharged from the facility.

While global health consensus supports the provision of PAC, its implementation in Tanzania has not been easy, despite strong efforts. Service oversight in the country is segmented between units within the Ministry of Health, Community Development, Gender, Elderly and Children (MOHCDGEC), with the Safe Motherhood Initiatives unit managing treatment of pregnancy complications and the Reproductive and Child Health unit overseeing contraception. The national PAC training curriculum includes contraceptive counseling, but it does not include method provision. Barriers to streamlined service oversight and limitations of national training tools have contributed to previous PAC programming focusing almost exclusively on emergency treatment (e.g., uterine evacuation) rather than a comprehensive package of curative and preventive services. Moreover, emergency obstetric care training and service provision tend to focus more on signal functions such as managing postpartum hemorrhaging rather than treating complications from an abortion—in part due to stigma—regardless of the gravity of both. Service segmentation within the facility also adds a logistical and privacy burden: PAC is based in the maternity ward, where postabortion clients interact with delivering mothers, while contraceptive counseling and provision are offered in facility family planning rooms. The absence of national health management information system (HMIS) tools for PAC has prevented the standardized documentation of services and limited the ability of facility and district health team management to use performance data for decision making.

With funding from an anonymous donor, Pathfinder International and MOHCDGEC implemented the *Chaguo La Maisha* (CLM) or “Choice of Life” project in January 2015 to strengthen and expand contraception services and PAC in public facilities of Dar es Salaam. CLM began in 36 facilities in 1 district in 2015 and eventually scaled to 96 facilities in 4 districts by 2018 for contraceptive services; PAC was introduced in 64 of these facilities. The CLM-supported PAC service package includes the use of manual vacuum aspiration (MVA) for uterine evacuation if indicated; provision of high-impact,^16^ nonjudgmental, and respectful youth-friendly services (YFS) (Box); comprehensive contraceptive counseling and expanded method mix provision at point of treatment; STI (including HIV) screening and treatment; follow-up appointment for continued care; and community linkages. This article describes the approach we used to introduce and expand PAC service coverage and demonstrates its effectiveness in removing barriers to accessing voluntary contraceptive services among postabortion clients.

Between 2015 and 2018, Pathfinder and MOHCDGEC strengthened and expanded contraception services and PAC in public facilities of Dar es Salaam.

BOX **Key Attributes of Pathfinder-Supported Youth-Friendly Postabortion Care Services**

- Providers receive training, values clarification, and mentorship to offer **nonjudgmental, respectful care** and the **full contraceptive method mix** regardless of age, marital, and matriculation status.
- Nonmedical staff receive training and values clarification to encourage an **enabling, welcoming environment** within the health facility.
- Service reorganization and infrastructure improvements ensure **confidentiality**, with audio and visual **privacy**.
- Service reorganization and contraception commodity collocation in PAC rooms ensure **24-hour per day/7-days per week availability** to meet schedules of young people.
- Community mobilizers sensitize on the availability of quality PAC and contraceptive services to build **community support**.
- Improved data collection instruments capture **age group disaggregation** among young people (preferably ages 10–14, 15–19, and 20–24).

## METHODS

### Baseline

In February 2015, Pathfinder International and the Regional Health Management Teams (RHMTs) conducted a baseline assessment of existing PAC and contraceptive services in 36 public facilities (4 hospitals, 2 health centers, and 30 dispensaries) in Temeke district of Dar es Salaam. The teams assessed the facilities with regard to client volume; service availability, organization, and integration; provider training; elements of YFS; and data collection/use practices. Key baseline results found that 12 facilities (33.3%) offered MVA for treatment of incomplete abortion (with most facilities using sharp curettage as the standard for treatment), 1 facility (2.8%) offered contraception post-procedure (pills only), and no facilities (0%) provided all components of PAC together (Table). Pathfinder, the RHMTs, and facility management used deficiencies found during baseline to create facility-based action plans and supported their implementation.

**TABLE.** Summary of Selected Results From the Baseline Assessment of PAC Among 36 Facilities in Temeke District, Dar es Salaam, Tanzania (February 2015)

**Element**	**Results**
PAC services	• 12 (33.3%) facilities offered MVA treatment for incomplete abortion.
	• No facilities offered all components of PAC.
	• 1 (2.8%) facility offered contraception (pills only) post-procedure.
	• No facilities had a dedicated private room for PAC.
	• 4 providers in 3 facilities had training in PAC.
	• 1 (2.8%) facility had PAC guidelines.
Commodities and equipment	• 8 (22%) facilities had ergometrine available.
	• 10 (27%) and 3 (8%) facilities did not have sodium hypochlorite or analgesics, respectively.
	• 32 (89%) facilities had either no or poor-quality MVA equipment.
	• 18 (50%) facilities had either no or poor-quality sterilization equipment.
	• 16 (44%) facilities had either no or poor-quality examination tables.
Registers	• No facilities had standardized registers, reporting tools, or procedures for documenting PAC.

Abbreviations: MVA, manual vacuum aspiration; PAC, postabortion care.

### Program Interventions

We supported a mutually reinforcing package of quality improvement interventions that encompassed provider clinical training and follow-up mentorship, including provision of YFS; service reorganization, supportive supervision, and equipment provision, including colocation of a range of contraceptive methods where PAC is offered; usage of a standardized PAC register that captures treatment of incomplete abortion type (e.g., MVA and sharp curettage), contraceptive method selection, and age disaggregation; and community engagement to increase awareness and acceptance of PAC and contraceptive services. (While PAC tools captured both MVA and sharp curettage, our training focused exclusively on MVA for the treatment of incomplete abortion and encouraged providers to shift away from the use of sharp curettage. Misoprostol is not used for treatment of incomplete abortion in Tanzania.) This article only includes MVA client data in the presented PAC results.

#### Provider Training and Follow-Up Mentorship

We applied a 2-phased approach of training and onsite follow-up mentorship to strengthen the clinical skills and confidence of PAC providers to comfortably deliver and document services. As of June 2018, we trained 236 providers in PAC, 120 of whom were midlevel providers (e.g., enrolled and registered nurses) in support of task-sharing efforts to increase the number of providers trained and authorized through delegation to provide MVA and contraceptive methods. We used the national training curriculum and added modules on values clarification and YFS orientation to reduce stigma and bias pertaining to women and adolescents who need reproductive health services and included content on counseling and the provision/removal of LARCs. All PAC providers received clinical training in MVA and contraceptive method (including LARCs) insertion and removal. LARCs provided under this program are Jadelle and Implanon implants and the Copper T 380A intrauterine device (IUD).

As of June 2018, we trained 236 providers in PAC, 120 of whom were midlevel providers.

Providers learned balanced counseling techniques to assess the client’s reproductive health intentions and medical eligibility prior to counseling on methods. Counseling reviews method options, starting with the most effective ones and progressing to less effective ones, as well as opportunities for method removal if desired.^17^ Research shows that a rights-based approach to contraceptive counseling should focus on method effectiveness as the leading factor, and Stanback and colleagues argue the following^18^:

the effectiveness of any contraceptive method is its paramount characteristic, and counseling that does not use [World Health Organization] tiers (with the most effective methods discussed proactively) fails to meet the true needs and desires of the majority of women.

YFS orientation included offering the full contraceptive method mix to adolescents and youth regardless of age, marital, and matriculation status. Finally, providers received no incentives for clients adopting contraception or specific methods, and service provision was not contingent upon clients accepting contraception.

We engaged 12 experienced providers from MOHCDGEC (to strengthen ministry personnel and support buy-in) and trained them to serve as mentors, using the ministry’s *Reproductive, Maternal, Newborn, Child, and Adolescent Health Clinical and Facility Mentorship* curriculum as part of the onsite follow-up post training. While all providers received some mentorship follow-up, the program specifically targeted individuals considered to have the most need of follow-up support. Mentors conducted mentorship in a range of clinical areas, including PAC. A tablet-based mobile application guided mentors through service steps using international and national guidelines and quality standards to track provider skill during service provision and over time. These checklists also guided mentors through verification of YFS components such as provider assurance of privacy, confidentiality, and judgment-free care. Under the PAC application, mentors monitored quality service indicators such as the providers’ ability to appropriately assess the client’s SRH intentions (including desire for future pregnancy), offer comprehensive contraceptive counseling (including opportunities for method removal if desired) and recommendation for the timely initiation of a method after abortion to protect from future unintended pregnancy or support appropriate spacing of pregnancies, and document services on client’s family planning card.

We engaged 12 experienced providers and trained them to serve as mentors to provide follow-up support to newly trained providers.

#### Service Reorganization and Equipment Provision

Prior to project intervention, only a few facilities offered multiple components of PAC (e.g., MVA and postabortion contraception), but did so in disparate parts of the facility and by different types of providers. Much of the onus for receiving comprehensive service fell on the client. She would often have to navigate various service rooms (e.g., maternity, family planning room, and pharmacy) and would encounter many providers and clients along the way, which compromised privacy and confidentiality and presented further barriers to service access.

We worked with the RHMTs, council health management teams, and facility managers to assess PAC service layout and organization and make appropriate upgrades. Our advisors stressed the importance of PAC being offered in a separate space if possible (as opposed to within the maternity), for optimal client privacy and confidentiality, and for the collocation of an expanded method mix of contraceptive commodities to encourage informed choice and voluntary uptake immediately post-procedure. The advisors advocated for PAC service availability 24 hours a day, 7 days a week and worked with facility managers to identify capacity support needed to ensure this schedule. We worked within existing facility layouts to provide targeted infrastructure improvements to increase the auditory and visual privacy and confidentiality of PAC. Finally, we worked with facility managers and supply chain staff to ensure the collocation of contraceptive commodities, other essential PAC commodities, and appropriate equipment within PAC rooms. By June 2018, among the 64 facilities, 40 provided PAC in separate rooms, 15 provided PAC in partitioned space within the family planning room, and 9 continued to provide PAC in the maternity ward (due to space limitations that prevented facilities from separating PAC from maternities, as advised). We equipped these facilities with a total of 380 MVA kits, 309 sterilization drums, 271 instrument trays, 130 IUD and implant kits for insertions and removals, 13 autoclave units, and other essential equipment for PAC.

We worked with the RHMTs, council health management teams, and facility managers to assess and improve PAC service layout and organization.

#### Standardized PAC Registers

The baseline assessment identified no standardized register for PAC as a major barrier to the documentation of services. The few facilities offering postabortion treatment used improvised counter books as registers. These registers were typically kept in the operating room (where contraceptive methods were not offered) and were frequently misplaced, leading to inconsistent use. Contraceptive counseling and method provision for postabortion clients were not documented alongside treatment services. While facility family planning registers identified postabortion contraceptive users, few postabortion clients were counseled and referred for contraception, and there was no process by which family planning service documentation was linked with postabortion treatment services provided elsewhere in the facility. Between July and December 2016, we developed a standardized PAC register with age group disaggregation for youth and adolescents (ages 10–14, 15–19, and 20–24) that tracked contraceptive counseling, method provision, and referral for STI treatment, among other quality indicators. With our support, facilities back-entered service data for the period of January to June 2016 and used the registers moving forward. Provider mentorship and supportive supervision visits reviewed registers and cross-checked them with HMIS data for accuracy and consistency. Finally, our advisors worked with district health teams and facility managers to strengthen routine performance monitoring of PAC and voluntary contraception services for decision making.

#### Community Engagement and Mobilization

To strengthen community awareness and access to health services, we recruited 215 community mobilizers to provide counseling and referrals for contraceptives and PAC. Community mobilizers received a 2-week training and orientation to a systematic door-to-door approach to identify, counsel, and provide respectful care to clients. During home visits, community mobilizers sensitized women to the availability and safety of PAC using mobile-based job aids. Between July 2015 and June 2018, community mobilizers counseled 283,127 women on contraception and PAC; generated 208,691 referrals (mostly for contraception), of which 96,702 were completed by the client; and provided 30-day follow-up visits to 112,686 women.^19^

### Data Collection and Analysis

We used national HMIS data generated from project-introduced facility PAC registers and descriptive statistics to measure and analyze MVA and contraception results with age and method disaggregation. Technical assistance visits afforded us the opportunity to review data quality between registers and HMIS data with facility staff, and data quality audits provided standardized scrutiny and capacity support for improving data quality. For mentorship data, we used an open-sourced mobile application (i.e., CommCare) to collect data and guide mentorship visits. Mentors followed the mobile application, which uses a quality checklist for service provision, to record provider performance during mentorship visits and calculate competency scores. We downloaded mentorship data from the CommCare server and used descriptive statistics to measure and analyze provider mentorship performance in key postabortion contraception indicators.

## RESULTS

### Increased Uptake of Voluntary Postabortion Contraception

**FIGURE 1:**
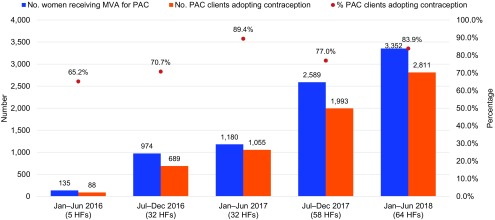
Total MVA and Postabortion Contraceptive Uptake Abbreviations: HF, health facility; MVA, manual vacuum aspiration; PAC, postabortion care.

**FIGURE 2:**
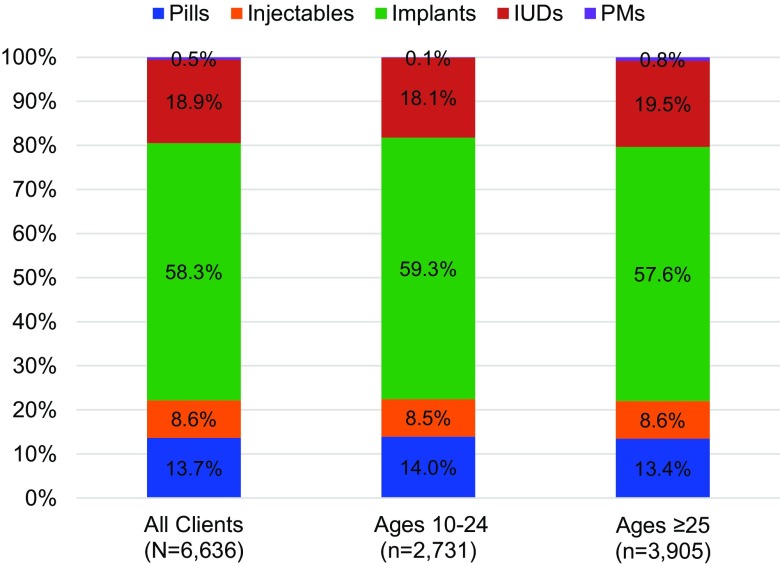
Method Mix Among Postabortion Contraceptors Abbreviations: IUDs, intrauterine devices; PMs, permanent methods.

From January 2016—when facilities started reporting PAC using a standardized register—to June 2018, uptake of voluntary postabortion contraception increased steadily as PAC coverage expanded from 5 to 64 facilities. The number of postabortion clients voluntarily choosing contraception before discharge from the facility increased each semester from 88 (5 facilities) in January–June 2016 to 2,811 (64 facilities) in January–June 2018, for a total of 6,636 clients during the 30-month period. The proportion of postabortion contraceptors among all MVA clients was 65.2% in January–June 2016 and 83.9% in January–June 2018 (Figure 1), for an average of 80.6% (6,636/8,230) during the 30-month period (not shown). Adolescents and youth (ages 10–24) accounted for 40.9% (3,364/8,230) of MVA clients and 41.2% (2,731/6,636) of postabortion contraceptors (not shown). Contraceptive method mix for all postabortion contraceptors (n=6,636) from January 2016 to June 2018 was 58.3% implant, 18.9% IUD, 13.7% pills, 8.6% injectables, and 0.5% permanent methods. Method mix for postabortion contraceptors was comparable between young people (ages 10–24) and adults (ages ≥25) (Figure 2).

Uptake of voluntary postabortion contraception increased steadily as PAC coverage expanded from 5 to 64 facilities.

**FIGURE 3:**
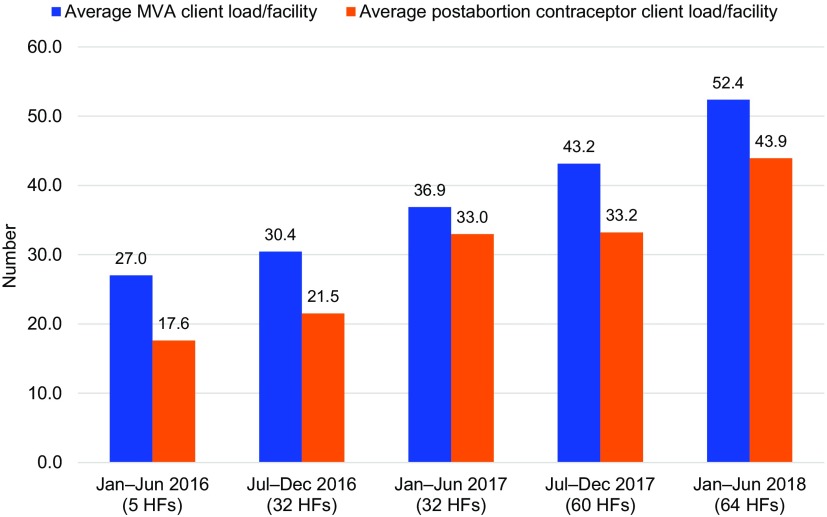
Average Semesterly PAC Client Load per Facility Abbreviations: HF, health facility; MVA, manual vacuum aspiration; PAC, postabortion care.

Coverage for PAC expanded, with both the number of facilities offering the service package and the average facility’s PAC service output increasing over time. To offset the effect of service expansion to new facilities on voluntary postabortion contraception performance, we calculated an average semesterly PAC client load per facility. Results show average MVA service use increased from 27.0 to 52.4 clients per facility per semester and average postabortion contraceptors increased from 17.6 to 43.9 clients per facility per semester between the first (January–June 2016) and last (January–June 2018) periods (Figure 3).

### Improved PAC Provider Capacity to Offer Contraceptive Services

**FIGURE 4:**
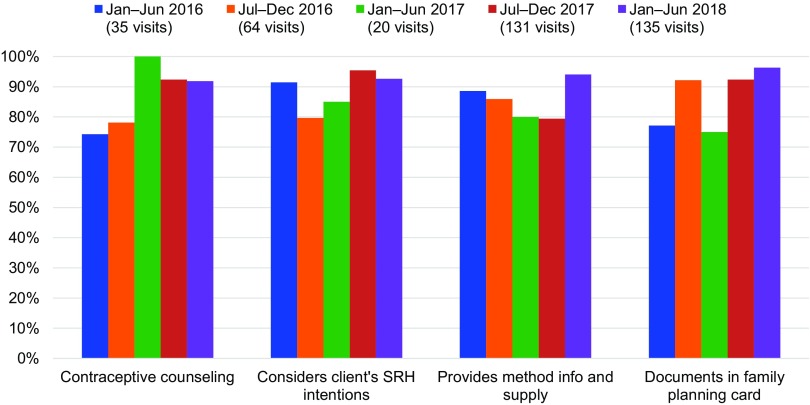
Average PAC Provider Performance on Key Contraceptive Indicators Abbreviations: PAC, postabortion care; SRH, sexual and reproductive health.

Through mentorship, providers received post-training follow-up support in PAC. Between January 2016 and June 2018, 12 mentors supported 161 providers during 385 observed PAC visits, for an average of 2.4 mentored PAC client visits per provider. Over time, mentored PAC client visits yielded improvements in provider capacity to offer voluntary contraceptive services to postabortion clients. The average percentage of mentored providers counseling clients on contraception and encouraging its timely initiation after abortion increased from 74.3% (January–June 2016) to 91.9% (January–June 2018), while those offering contraceptive method information and supply prior to client discharge increased nominally from 88.6% (January–June 2016) to 94.1% (January–June 2018). The average percentage of mentored providers who considered the client’s SRH intentions, including desire for future pregnancy, did not change significantly, at 91.4% (January–June 2016) and 92.6% (January–June 2018). Service documentation, however, improved among mentored providers: the average percentage of providers documenting service details in the client’s chart and family planning card increased from 77.1% (January–June 2016) to 96.3% (January–June 2018) (Figure 4).

Mentored PAC client visits yielded improvements in provider capacity to offer voluntary contraceptive services to postabortion clients.

**FIGURE 5:**
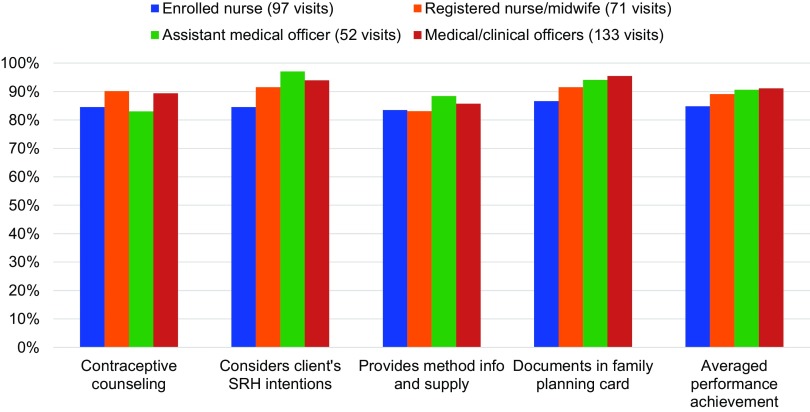
Average PAC Provider Mentorship Performance on Key Contraceptive Indicators, by Provider Cadre Abbreviations: PAC, postabortion care; SRH, sexual and reproductive health.

While overall PAC provider capacity to offer contraceptive services improved over time, performance across provider cadre was comparable. Mentorship supported enrolled nurses, registered nurses/midwives, assistant medical officers, and medical/clinical officers to strengthen the quality of PAC as we expanded support to more hospitals, health centers, and dispensaries. Different provider types performed within 7 percentage points (from 83.1% to 90.1%) for offering contraceptive counseling and 5.3 percentage points (from 83.1% to 88.4%) for providing method information and supply. Provider cadres performed with slightly more variation for considering the client’s SRH intentions and documenting services on the client’s family planning card, within 12.6 percentage points (from 84.5% to 97.1%) and 8.9 percentage points (from 86.6% to 95.5%), respectively. Overall, provider cadres performed within 6.3 percentage points (from 84.8% to 91.1%) when achievement was averaged across all 4 service quality indicators (Figure 5).

## DISCUSSION

Abortion rates in Tanzania’s eastern zone (which includes Dar es Salaam) are high, estimated at 23.9 per 1,000 live births in 2013.^20^ During this time, an estimated 54,655 induced abortions occurred per year for the region, many of which went without proper care: for every woman treated for an induced abortion, more than 8 women did not receive treatment. In an environment where abortion is legally restricted and highly stigmatized and access to PAC is significantly limited—with fewer than 8 facilities providing the service per 100,000 women nationally^20^—many women seek unsafe abortions and do not receive quality treatment for resulting complications. Compounding these problems, access to contraception is also limited, with only 48% of the demand for modern method contraception being met in Dar es Salaam.^3^ Contraception is well accepted and in high demand among women in Dar es Salaam admitted with complications from an unsafe abortion,^21^ yet postabortion clients have traditionally faced obstacles in accessing contraception. These obstacles may place them at heightened risk of having future unintended pregnancies and seeking additional unsafe abortions.

In this context, we strengthened and expanded PAC coverage to primary- and secondary-level public facilities across 4 districts in Dar es Salaam. We increased the number of facilities offering PAC and using a standardized PAC register from 0 at baseline to 64 through June 2018, 48 (75%) of which were at the primary (dispensary) service level. Of the 64 facilities offering PAC, 64 (100%) offer contraception at point of treatment, 24 hours per day/7 days per week, and 55 (86%) offer PAC in a private space exclusively used for that service. Comprehensive counseling and method mix availability at point of treatment improved, from 1 facility offering 1 short-acting method at baseline to 64 facilities offering at least 5 modern methods, including LARCs and voluntary sterilization. Evidence has shown that improving the quality of counseling and expanding the range of method options available to women promote informed and voluntary choice.^13^ We increased the number and breadth of provider cadres eligible to offer PAC, training 236 providers, including 120 midlevel staff such as enrolled and registered nurses. Finally, communities improved their awareness of and linkage to PAC through a network of community mobilizers.

Service results demonstrate that these efforts had a significant effect on women accessing comprehensive contraceptive counseling and provision. In a 30-month period, 6,636 women, who previously might not have had the opportunity, increased their protection from future unintended pregnancies and possible additional abortions through uptake of voluntary postabortion contraception. Facilities increased their average semesterly client load as more people learned about and accessed PAC, with use of MVA for PAC nearly doubling and postabortion contraception increasing 2.5-fold between the first and last periods. The postabortion contraceptive uptake rate was high, at 80.6% between January 2016 to June 2018, which surpasses the recommended quality benchmark of at least 60% of all women receiving abortion services accepting contraception^22^ and is in line with other regional experiences.^14^^,^^23^^,^^24^

Service results demonstrate that our efforts had a significant effect on women accessing comprehensive contraceptive counseling and provision.

Mentorship results show PAC provider capacity for quality contraceptive indicators improved over time and was comparable across provider cadres. Research has identified important subthemes in quality abortion care indicators, including those that measure informed decision making by the client.^10^ We found that PAC providers performed well for related indicators, with improvements in their ability to effectively counsel women in contraception, provide method information and supply, and document services, while maintaining high capacity to consider clients’ SRH intentions—including desires to become pregnant. Through task-sharing efforts, we expanded the cadre of medical personnel qualified to offer PAC to include staff who traditionally did not offer surgical services such as MVA. Evidence shows that expanding PAC—including MVA—to midlevel providers who traditionally offer contraception at primary health facilities helps to expand access to quality PAC and supports uptake of postabortion contraception.^25^ However, while some research has found that provider type can influence access to and uptake of postabortion contraception,^26^ we found contraceptive service offering was relatively comparable across different PAC provider cadres, with the average performance for 4 quality service indicators falling within 6.3 percentage points across 4 groups of providers. We found an expanded cadre of PAC provider types successfully increased coverage of postabortion contraception, with minimal variance in contraceptive service quality.

Method selection results are notable, with 77.3% (5,127/6,636) postabortion contraceptors adopting LARCs before discharge from the facility. Adults and young people (ages 10–24) had comparable preference for LARCs: 77.2% adults (3,013/3,905) and 77.4% young people (2,117/2,731) voluntarily selected LARCs. Immediate contraceptive service offering is crucial for helping women avoid risk of future unintended pregnancy because fertility can return as soon as 8–10 days postabortion. Studies have demonstrated that the adoption of a LARC immediately post-procedure protects a woman more from subsequent unintended pregnancies and repeat abortions than if it is adopted 3–6 weeks post-procedure.^13^ Demand for LARCs among postabortion clients has been shown to increase substantially when providers and facilities are skilled and equipped to offer them,^15^ and our experience supports this claim. Nonetheless, the LARC adoption rate was higher than seen in similar programs. We worked to increase access to and availability of LARCs in postabortion and standard contraceptive services and informed clients of options for method removal through several mutually supporting interventions, such as clinical training and mentorship in LARC insertion and removal and YFS to a broader range of health providers, equipment provision (e.g., IUD kits), service integration, and community referrals. A focus on balanced counseling and no provider incentivization reinforced voluntarism and informed choice among clients.

More than three-quarters of postabortion contraceptors adopted LARCs before discharge from the facility.

### Challenges

MOHCDGEC was an essential partner and much of CLM’s success hinged upon its leadership, technical capacity, and support. Despite these advantages, the organization of technical units within the ministry did not always lend itself to strengthening PAC. Different ministry technical units manage different service components of PAC (e.g., treatment and contraception), which presents a segmented package of services. It was challenging at times to reinforce a service package that spanned different technical units, health care providers, and service registers, and required Pathfinder, district health teams, and facility management to work across structural divides to build PAC.

Limitations to facility space and our mandate to make improvements presented obstacles to reorganizing PAC in separate designated rooms in each facility. Where creating a PAC room was not feasible, we worked with facilities to partition a private space for PAC within the family planning room (15 of 64 facilities). Yet sometimes even this arrangement was not possible, particularly in smaller dispensaries. In these cases (9 of 64 facilities), we worked with facilities to offer PAC within the labor and delivery room.

While we strengthened PAC service documentation through introducing standardized reporting tools and providing capacity support to manage and use quality data, evidence shows that more support is needed to ensure all services are adequately documented. Historically, some providers offered postabortion treatment under the table to make supplemental income, taking advantage of confusion and poor transparency regarding services. Our work to mainstream PAC, including increased oversight by facility managers and improved provider confidence to document services, helped curb this shadow practice. Yet, we still found in selected cases, facilities required MVA kit replacements at rates faster than expected in comparison to documented service delivery, suggesting more kits were being used than what the registers showed. In these instances, we engaged facility managers and providers to address the issue and provided refreshers on service documentation and data review practices.

While Pathfinder strengthened PAC service documentation, more support is needed to ensure all services are adequately documented.

### Limitations

Limitations of this study include the absence of an established control group for comparison with the results. Every facility involved was an intervention site because resource limitations did not permit data collection in comparison facilities that had not received our support. Similarly, while baseline data afford a basic understanding of what the service quality and level were prior to the intervention, treatment data were sporadic and postabortion contraception data were largely unavailable before we began the program because facilities did not use standard and comprehensive logbooks for services. This circumstance prevents a true comparison of postabortion contraception data before and after the intervention.

Mentorship data documenting provider–client interactions during service delivery are useful for adding depth of quality to general service statistics, yet they pose limitations nonetheless. The information covers only a portion of PAC provided and varies in provider representation because the intervention’s focus was to improve the quality of providers thought to most need it. Furthermore, mentors covered a range of SRH services during their visits, including postpartum and general contraception provision. The sporadic nature of uptake of PAC, with 27–52 clients accessing care on average per facility each semester, made it difficult to always align service provision with planned mentorship visits. Therefore, mentorship data represent only a fraction of PAC provided (approximately 5%), and it is difficult to fully compare this data set with general PAC service statistics.

Finally, another limitation was the lack of a systematic process for effectively monitoring method discontinuation among postabortion clients. We trained and mentored providers to remove LARCs, and community mobilizers followed up with postabortion clients who were referred for PAC service, but no system or tool existed to comprehensively track postabortion contraceptors after discharge. Ascertaining how many clients discontinued their method over time was not possible, despite method interruption being a common reality faced by programs. Makensius and colleagues found that 25% of postabortion contraceptors in Kisumu, Kenya, discontinued their method within 3 months, with LARCs accounting for the most commonly discontinued method type.^27^ Our results do not show method discontinuation, which represents a gap in the data.

## CONCLUSIONS AND RECOMMENDATIONS

Introducing and expanding coverage of PAC—particularly at lower levels of the health system—form a clear strategy for removing barriers to accessing postabortion contraception and suggest a path toward reducing rates of unintended pregnancies and unsafe abortions. Our experience reinforces evidence regarding global recommendations for PAC, including recommendations intended to ensure adolescents and youth access quality services. The program approach described here touches upon several essential and interrelated health systems strengthening and service delivery components and includes the following:

- Clinical PAC training and follow-up mentorship—including MVA, contraceptive counseling and provision, values clarification, and YFS—for mid- and senior-level provider cadres to ensure they apply learned clinical skills consistently and with confidence and respect
- PAC service organization and equipment supply to encourage privacy, confidentiality, and streamlined services (e.g., separate space for PAC; service availability 24 hours per day/7 days per week; and collocation of an expanded method mix and other PAC commodities)
- Standardized PAC tools with disaggregation for client age group (ideally 10–14, 15–19, 20–24, and ≥25 years), treatment type, and method selection
- Community engagement to increase awareness and acceptance of, and linkage to, facility-based gratis PAC

While implementing this package of support helped to strengthen the quality and expand coverage of PAC across a network of low- and mid-level health facilities, the process was time and resource intensive. We adapted its approach over time to improve feasibility of bringing interventions to scale and lay foundations for sustainability. Accordingly, we recommend a few strategies:

- Utilize and build upon technical leadership within ministries of health, particularly at regional and/or district levels, for leading provider capacity building efforts, including clinical training and mentorship
- Integrate "treatment of postabortion complications with contraceptive service programming to ensure an appropriate investment and stable supply chain of contraceptive methods
- Implement on-the-job training with low dosage, high frequency rather than large one-time off-site trainings for improved skills and knowledge retention and reduced cost
- Invest in building capacities of facility managers through mentorship and supportive supervision, including service organization and data usage, to foster ownership and investment in quality PAC
- Support and strengthen a culture of performance review and accountability within the health facility, including regular data reviews and action planning/follow-up

Although PAC has been on the global agenda for years and consensus surrounds the high-impact practice of postabortion contraception, momentum in expanding the service package has only recently been made in Tanzania. Nonetheless, clear indicators of progress exist. In early 2019, MOHCDGEC approved official PAC data collection tools and is currently rolling them out across the health system. These tools will feed critical data into the national HMIS and will enable ministry officials, facility managers, and providers to monitor services appropriately. The “focus on implementation and use of data to drive coverage, especially when interventions are delivered at lower levels of the health system,” is a critical step toward reducing maternal mortality.^4^ Yet Tanzania has more work to accomplish to address key bottlenecks to expanding PAC coverage, such as service segmentation within the ministry and facility physical spaces, human resource constraints, and the prevailing confusion about the legality and stigmatization of PAC. Sustained investment (both nationally and internationally) and commitment to support expansion of PAC to lower facility levels will be critical steps for Tanzania to take in its effort to increase access to contraception and reduce maternal mortality.

## Notes

**Peer Reviewed**

**Funding:** Anonymous.
